# Determination of Correlated Color Temperature in Ex Vivo Porcine Eyes during Intraocular Illumination

**DOI:** 10.3390/jcm12083034

**Published:** 2023-04-21

**Authors:** Nicole Fehler, Martin Hessling

**Affiliations:** Institute of Medical Engineering and Mechatronics, Ulm University of Applied Sciences, 89081 Ulm, Germany

**Keywords:** correlated color temperature, ophthalmology, diaphanoscopy, endoillumination, intraocular illumination

## Abstract

(1) Background: In ophthalmic surgery, white light is mostly applied to illuminate the intraocular space, and ophthalmologists are comfortable working with it. Diaphanoscopic illumination changes the spectral composition of light, resulting in a change in the correlated color temperature (CCT) of the intraocular illumination. This color change makes it difficult for surgeons to recognize the structures in the eye. CCT during intraocular illumination has not yet been measured before, and it is the aim of this study to perform such measurement. (2) Methods: CCT was measured inside ex vivo porcine eyes during diaphanoscopic illumination and endoillumination using a current ophthalmic illumination system with a detection fiber inside the eye. By applying pressure on the eye with a diaphanoscopic fiber, the dependency of CCT on pressure was examined. (3) Results: The intraocular CCT values during endoillumination were 3923 K and 5407 K for the halogen and xenon lamps, respectively. During diaphanoscopic illumination, a strong unwanted red shift was observed, resulting in 2199 K and 2675 K for the xenon and the halogen lamps, respectively. Regarding different applied pressures, the CCT did not differ considerably. (4) Conclusions: This red shift should be compensated for in the development of new illumination systems since surgeons are used to white light illumination, which also simplifies the identification of retinal structures.

## 1. Introduction

For illuminating the interior of the eye during ophthalmic surgery, a variety of light sources are available. In addition to halogen and xenon lamps, the application of LEDs is increasing. Due to the spectral differences in the emission spectrum, their correlated color temperature (CCT) is also different.

**Figure 1:**
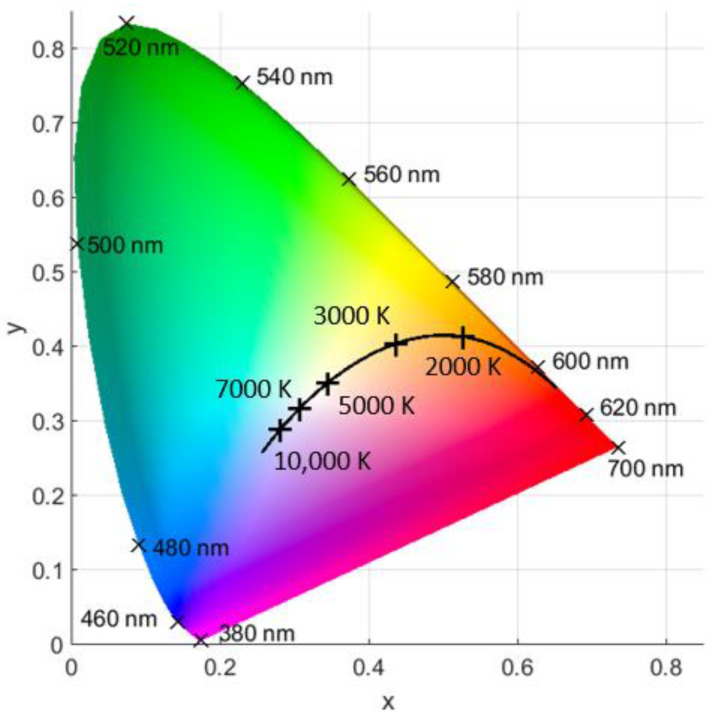
The CIE 1931 color space chromaticity diagram with inserted black body curve (black line) and markers for colors of different wavelengths.

CCT describes the temperature at which a hypothetical black body would have to be heated to emit the same color as the light source. A high CCT appears bluish white and is known as cold CCT, whereas a low CCT is called warm and appears reddish. This is illustrated in the CIE (Commission Internationale de l’éclairage) 1931 color space chromaticity diagram in Figure 1.

Halogen lamps are the most suitable choice at the beginning of a 20-gauge vitreous surgery and usually have a warm white CCT of around 2600 K [1], which is lower than the typical CCT of 4500 K of xenon lamps [2]. Xenon lamps emit a more bluish white light, but this increases the hazard to the retina at the same brightness of the light source. This also applies to white LEDs, which are recently used for ophthalmic surgery. CCT depends on the LED type. LEDs with a lower blue content in their emission spectrum are warm white LEDs and exhibit a low CCT (around 2700 K), whereas cold white LEDs have a high CCT (up to 6500 K) [1,3].

With higher CCT, the emission spectrum of a lamp tends to have a higher blue component. However, with increased blue content in the emission spectrum, the hazard to the retina increases. Blue-light hazard is higher for higher CCT than for lower CCT [4,5]. The exposure time during surgery can be extended by more than a factor of two by using a white LED with 3000 K instead of a 6500 K white LED [6]. A study in 2021 investigated the effect of LEDs with different CCTs on retinal cells and found that the viability and number of retinal cells decreased with increasing CCT [7]. In 2020, it was also published that white LED light with higher CCT was more likely to cause retinal injury in mice than white LED light with lower CCT [8]. However, CCT should not be considered a decisive measure of potential damage to the eye without information on intensity. The relevant value for retinal risk is irradiance and its spectral distribution.

Depending on the CCT, structures can be distinguished better or worse. For example, it has been found that the ability to identify veins depends on the white-light setting [9] and is better when using an illumination setting with a higher CCT than 2700 K [10]. The spectral distribution of light and, thus, the CCT of the illumination light also influence imaging in bronchoscopy, oral examinations, or renal examinations [11,12,13]. Analogous to these biological tissues, visualization of the interior of the eye is also related to the spectral intensity distribution and the CCT of the illumination light used. Illuminating the eye with a more reddish spectrum (low CCT) improves the visibility of indocyanine green stain, whereas reducing the red portion of the spectrum (higher CCT) improves the visibility of the vitreous body [14]. Images taken with light with a high red content (small CCT) result in supersaturated and potentially less informative retinal images, whereas images taken with light with a broader gamut are more balanced and feature a greater color discrimination, which is important, for example, in diabetic retinopathy [15]. Mundinger et al. (2019) conducted a study in which surgeons evaluated the color appearance of internal anatomy under lightning with different CCTs. The smallest CCT of 3000 K was rated as less effective compared to 4000 K, 4500 K, and 5000 K [16]. For further development of diaphanoscopic light sources, it would be desirable to be able to change the spectral distribution and, with this, the CCT of the illumination light used, so that surgeons can adjust the CCT for different surgeries. Mundinger et al. (2019) and other surgeons stated that they needed to learn more about the benefits of spectrally tunable lights [16]. Therefore, there is a need to examine different CCTs of different light sources with different applications on the eye (diaphanoscopy or endoillumination), which is the aim of our study.

With transscleral illumination, the spectrum of the light arriving in the interior of the eye changes due to the absorption properties of the eyewall. Long-wavelength red light penetrates the eye better than short-wavelength blue light [17] due to the high absorbance of hemoglobin and melanin in the low wavelength range [18]. Therefore, the intraocular CCT will also change with the transscleral application compared to the CCT of the illumination light used. In this study, this change in CCT is investigated.

In diaphanoscopic illumination, a surgeon can press the illumination fiber lightly or even more strongly against the eye. Previous studies have revealed a change in the spectral transmission properties of the eyewall, especially the sclera, as a result of the compression of the eyewall, allowing more light to enter the eye [17,19,20,21,22]. Therefore, CCT is examined for its pressure dependence in our study.

The CCT behind the sclera with the use of transscleral illumination was investigated by Hessling et al. 2015 [23]. However, CCT was only estimated based on the transmission property of the sclera. The retina and the choroid, which have high absorption in the low wavelength range, were not considered in their calculation.

In our study, the CCT inside ex vivo porcine eyes illuminated with two different ophthalmic illumination systems are determined during endoillumination and during diaphanoscopic illumination. The dependency of CCT on the applied pressure with the diaphanoscopic illumination fiber on the eye is also investigated. To our knowledge, no measured CCT values of this kind have been published so far.

## 2. Materials and Methods

**Figure 2:**
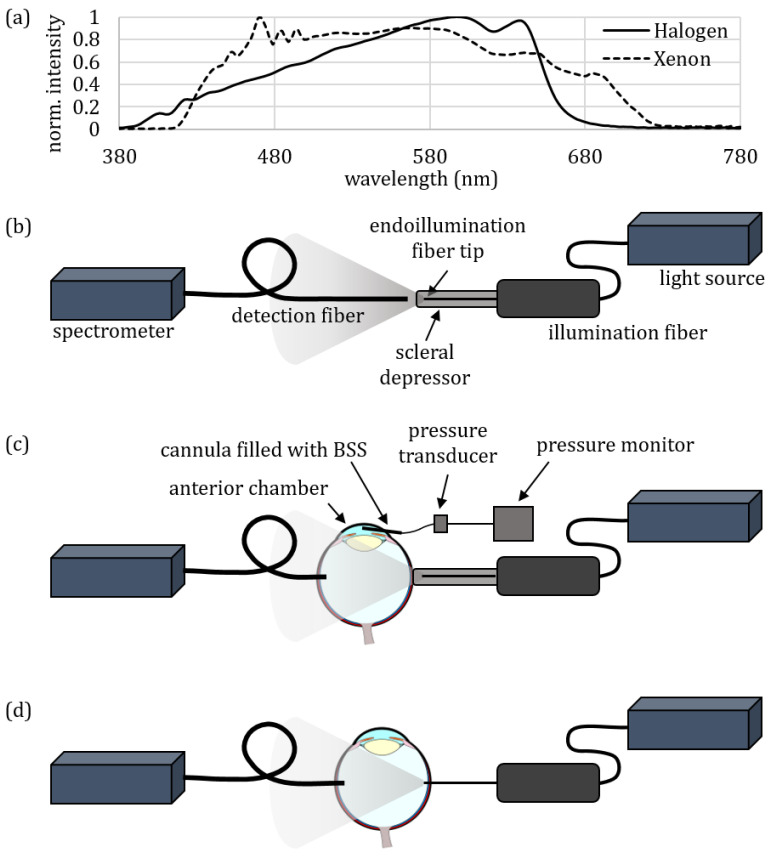
(**a**) Normalized intensity distribution of the halogen light source (solid line) and xenon light source (dashed line). (**b**) Set-up for determining the CCT of the lighting systems. (**c**) Set-up for determining the CCT during diaphanoscopic illumination and for investigating its pressure dependency. (**d**) Set-up for determining the CCT during endoillumination.

The measurements in this study were performed on ex vivo porcine eyes, since porcine eyes are anatomically and physiologically very similar to human eyes and, therefore, considered to be good alternative to human ones [24,25,26,27,28,29]. The porcine eyes were obtained from a local abattoir. The eyes were cleaned from surrounding tissues and stored at 8 °C in a balanced salt solution (BSS) in a refrigerator. The measurements were performed on the same day of enucleation. As an exclusion criterion, only eyes with a crystal-clear cornea (no sign of haze) were included in this investigation. The eyes were also excluded if no light transmission through the eyewall could be detected when looking through the lens while holding the eyeball against sunlight because of suspected hemorrhage due to the removal procedure in the slaughterhouse. CCT was determined for illumination with two different ophthalmological light sources: the halogen light source was an Accurus Surgical System version 600 DS (Alcon Laboratories Inc., Fort Worth, TX, USA), and the xenon lamp was a Xenon BrightStar (D.O.R.C., Zuidland, The Netherlands). The normalized emission spectra of these light sources are illustrated in Figure 2a. As the illumination fiber, an ophthalmological light guide, TotalView Endoillumination Probe, including an illuminated scleral depressor (23 gauge) (D.O.R.C., Zuidland, The Netherlands) was used. The halogen lamp was set to 100% intensity and the xenon lamp was set to 50% intensity, which was the highest possible intensity that could be selected in combination with this 23-gauge fiber. The initial CCT of the illumination light sources were measured as illustrated in Figure 2b. The illumination fiber tip was placed in front of the detection fiber with a diffusing spherical fiber tip IP85 (Medlight, Ecublens, Switzerland), which was connected to a spectrometer, AvaSpec-HSC 1024 × 58TEC-EVO (Avantes, Apeldoorn, The Netherlands). During transscleral illumination, the scleral depressor was attached to the fiber (Figure 2c), whereas during endoillumination, the depressor was removed so that the fiber could be inserted into the eye (Figure 2d). To insert the detection fiber into the eye, a cannula with 1.8 mm diameter was inserted in the equatorial region. For endoillumination, a trocar with 0.75 mm diameter was inserted on the opposite side of the eye, also in the equatorial region. For diaphanoscopic illumination, the last step was skipped. Then, the eye was placed on an eye holder with the anterior side up so that it was freely accessible and possible to look into the interior of the eye. The detection fiber was 0.85 mm in diameter and was introduced in the eye via a cannula with 1.8 mm diameter. The tip of the detection fiber was inserted 4 mm inside the eye. For diaphanoscopic illumination, the scleral depressor was brought in contact with the eyewall without applying force to the eye, which was then illuminated transsclerally. For endoillumination, the fiber tip was inserted through the trocar into the eye until the complete fiber tip had passed the eyewall and reached the interior of the eye (visible through the pupil). The set-up was calibrated with a calibration lamp CL2 (Bentham Instruments Limited, Reading, UK) according to the manufacturer’s specifications. To obtain the CCT, the spectrum was imported into the software Color Translator & Analyzer (CT&A) from BabelColor (The BabelColor Company, Montreal, QC, Canada), which displays the CCT. For endoillumination, 24 eyes, and for diaphanoscopic illumination, 25 eyes were used to determine the CCT. The dependency of CCT on the applied pressure of the illumination fiber on the eye was investigated analogously to the set-up described above. When pressing the illumination fiber onto the eye (Figure 2c), the pressure in the anterior chamber increases. These pressures were determined with a pressure transducer, Combitrans Monitoring-Set (Braun, Melsungen, Germany), using a cannula inserted into the anterior chamber of the eye, which was filled with a BSS. This cannula had to be inserted into the anterior chamber prior to the measurement. The transducer was connected to a monitor, SMK 231 (Hellige, Freiburg, Germany), which displays the pressure changes in the anterior chamber. The pressure increased when the diaphanoscopic illumination fiber was pressed against the eyewall. The CCT was determined for 3 different pressures in the anterior chamber, namely 23, 78, and 132 mmHg, in a total of 105 eyes for the halogen lamp and 18 eyes for the xenon lamp. By applying a force on the eyewall, the intraocular pressure in the anterior chamber increases, according to the correlation reported in [30]. To generate a consistent force on the eyewall, the intraocular pressure was measured because each eye was slightly different in its anatomy. For the smallest pressure of 23 mmHg, the illumination fiber was just in contact with the eyewall. At 78 mmHg, the fiber was also in contact with the eyewall but was pressed against it with a higher pressure so that the eyewall was somewhat depressed. At 132 mmHg, the eyewall was strongly depressed with the diaphanoscopic fiber. These values were selected according to previous studies [17,22,31].

## 3. Results

**Figure 3:**
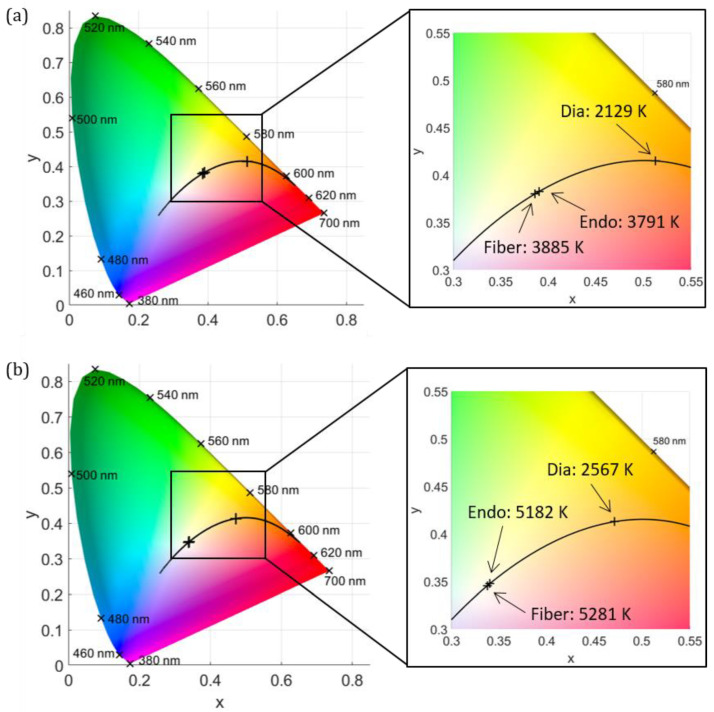
Comparison of CCT when illuminating the eye with a halogen lamp (**a**) and a xenon lamp (**b**). The CIE 1931 color space chromaticity diagram is illustrated (**left**) along with a zoom of the selected area (**right**). The CCT of the fiber emission (Fiber), the CCT inside the eye during endoillumination (Endo), and the CCT during diaphanoscopic illumination (Dia) are displayed.

The mean CCT values of the halogen and xenon lamps, with the corresponding standard deviations, are presented in Table 1, as well as the CCT values inside the eye during endoillumination and diaphanoscopic illumination. The CCT in the eye with endoillumination is approximately the same as without endoillumination. However, with diaphanoscopic illumination, the CCT drops considerably. The original cool white and warm white illuminations experience a red shift. This red shift is illustrated in Figure 3. In (a), the results of the halogen lamp, and in (b), the results of the xenon lamp are displayed. On the left side is the CIE 1931 color space chromaticity diagram, and on the right side, an enlargement of a selected area of the left diagram is illustrated, with the highlighted CCT of the fiber tip (Fiber), the CCT inside the eye during endoillumination (Endo), and the CCT during diaphanoscopic illumination (Dia).

**Figure 4:**
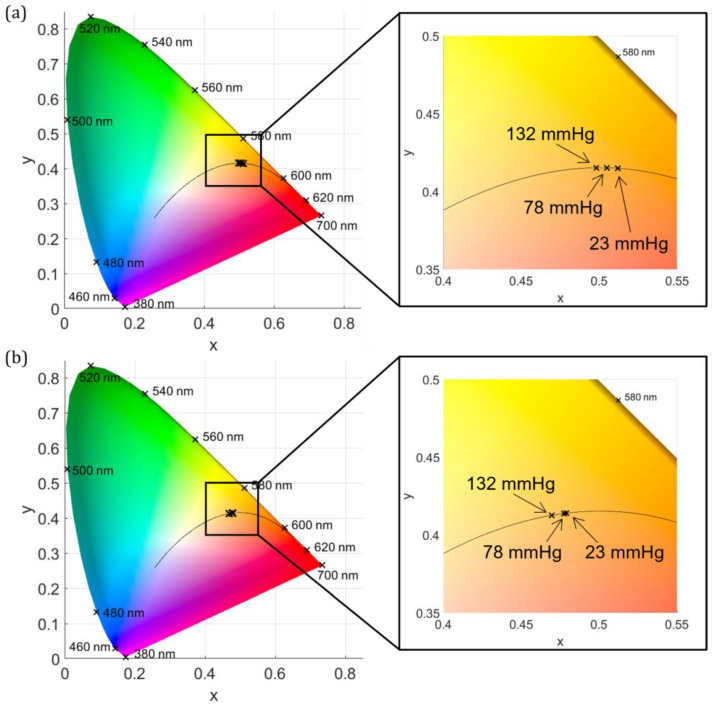
The CIE 1931 color space chromaticity diagram is illustrated (**left**) along with a zoom of the selected area (**right**). The CCT inside the eye during diaphanoscopic illumination is displayed for different applied pressures (23, 78, and 132 mmHg) for the halogen lamp (**a**) and the xenon lamp (**b**).

For the pressure-dependent measurements, the CCT increases with increasing pressure on the eye. For the measurements with the halogen lamp and with 23 mmHg, the CCT is (2136 ± 208) K; with 78 mmHg, it is (2206 ± 125) K; and with 132 mmHg, the CCT is (2275 ± 139) K. These results are illustrated in Figure 4a. Using the xenon lamp, the CCT rises from (2478 ± 156) K for 23 mmHg to (2494 ± 219) K for 78 mmHg and to (2586 ± 129) K for 132 mmHg, as shown in Figure 4b. The CCT increases slightly with increasing pressure but has a very high standard deviation. Therefore, an ophthalmologist would not be able to control the color of the illumination in the eye by applying these pressures to the eye with a diaphanoscopic illumination fiber.

## 4. Discussion

With diaphanoscopic illumination of ex vivo porcine eyes, the correlated color temperature inside the eyes experiences a relatively strong red shift compared to outside the eye. This red shift is due to the absorption properties of the eyewall, which contains a lot of hemoglobin and melanin, therefore allowing red light to pass through the eyewall better than blue light [17,19,32,33]. Since surgeons are accustomed to light with high CCT, this red shift complicates the identification of retinal structures [15]. Therefore, color temperature is a non-negligible property of ophthalmic illumination light sources for surgeons.

A comparison of porcine and human eyes reveals a lower melanin content in human eyes than in porcine eyes [34,35], which leads to a lower red shift of light since less blue light is absorbed in human eyes than in porcine eyes. Besides this difference between porcine and human eyes, there are many similarities. The porcine retina and its layers and capillaries are very similar to those of human eyes and are considered a suitable model for retinal studies [26]. The cone-rich area in the center of the porcine fundus has a similar function as a region of increased visual acuity in the human fovea [29]. The porcine retina has some similarities to the human photoreceptor pattern, including a non-tapetal fundus with a holangiotic vascular pattern and retinal layers of similar thickness [29]. The proportion of rods and cones is similar as well as the paramacular density of the cones [25]. De Schaepdrijver et al. (1992) also concluded that the porcine eye is a useful model for ophthalmic research on retinal and choroidal vasculature [28]. The sclera also has similar histology and collagen bundle organization, being a bit more disordered in the porcine eye, but the porcine sclera is about twice as thick as the human one [24]. However, the transmission properties of the sclera are similar [17,36]. The results of this study are limited to the porcine eye, but due to the facts mentioned above, we consider the porcine eye as a good model for the human eye.

Besides the disadvantage of the red shift of light in diaphanoscopic illumination compared to endoillumination, there are also advantages. Diaphanoscopy is a non-invasive method that requires a less sterile environment than endoillumination, which requires an incision into the eye, and with this, the risk of postoperative infection is lower. There are no unwanted reflexes at the lens when illuminating the fundus [37], and the transmitted light is homogenized by the scattering in the sclera, which results in a more diffuse incident illumination and, thus, shadows are reduced. The transmission properties of the eyewall also play a major role, ensuring that the risk to the retina is much lower than with endoillumination. Transscleral illumination can be useful in tumor localization [38,39,40], tumor therapy [41,42], diaphanoscopically controlled cyclophotocoagulation [43], removal of vitreous [44,45], peeling of ILM (internal limiting membrane) [46], and localization of retinal breaks [47,48,49].

When applying different pressures to the eye with the illumination fiber, the CCT increases with increasing pressure. However, this is not a significant effect, except between 23 and 132 mmHg. All values are in the warm CCT range and would appear relatively equally red to a surgeon, which can be seen in Figure 4. The difference in CCT between 23 and 132 mmHg is 139 K for the halogen lamp and 108 K for the xenon lamp. These values are in the range of the standard deviation of the respective mean values. Therefore, pressure dependence is not that important for CCT in ophthalmic surgery.

For the enhancement or development of diaphanoscopes, it is important to know the CCT a surgeon is used to during diagnosis and surgery. With this knowledge, new illumination systems can be developed with a spectral distribution that provides the desired intraocular CCT even with diaphanoscopic illumination. Therefore, it is beneficial to know that when using transscleral illumination, the CCT undergoes a red shift of about 1722 K (23 mmHg) and 1610 K (132 mmHg) for the halogen lamp and about 2803 K (23 mmHg) and 2695 K (132 mmHg) for the xenon lamp. New devices should be developed to counteract this red shift during diaphanoscopic illumination.

For determining the CCT of halogen lamps, their intensity is important. As the emission spectrum is shifted toward lower wavelengths with increasing intensity of the light source, the CCT also increases. We tested this behavior with the halogen lamp used in this study and could verify that the color temperature at 10% intensity is 2884 K and increases to 3558 K at 100% intensity. Thus, the CCT can be adjusted by varying the intensity of the halogen lamp. However, as the intensity of the light source decreases, so does the luminosity inside the eye, especially with transscleral illumination. The CCT of the xenon lamp does not depend on the intensity of the light source, whereas for LEDs, for example, the CCT decreases because the emitted spectrum undergoes a red shift with increasing intensity.

The CCTs determined in this study are only valid for the light sources used in this study and not for all halogen or xenon lamps, since CCT depends strongly on the emission spectrum of the light source. As many ophthalmic light sources contain a filter for the blue wavelength region, and some for the longer wavelength region, CCT could change a lot for different light sources. CCT might differ drastically for widely used LED illumination systems. A cold white LED has a much higher CCT than a warm white LED, which is also due to the different emission peaks of the LEDs. With the continuous development of LED technology, their spectral properties are also changing, leading to LEDs with a high CRI (color rendering index). The intraocular CRI during endoillumination and diaphanoscopic illumination is equally high, with a value up to 90 (measured analogously to CCT and the value is estimated by the software). Comparing the CRI for the halogen and xenon lamps reveals no differences. Since there are no differences due to light source or illumination method, the results are not presented here. The absorption of light in the eyewall apparently has no effect on the CRI of the light inside the eye. We also investigated the effect of the angle of illumination on CCT and observed no substantial influence of the angle on the CCT. Therefore, the results for this investigation are not presented.

During endoillumination, the CCT does not change. Light appears equally white inside the eye as it does outside the eye, which facilitates the intraocular vision for a surgeon. Analogous to endoillumination, it would be desirable to have a high CCT for diaphanoscopic illumination in order to better distinguish small structures. When new lighting systems are designed, filters are often installed to filter out short-wave light. For example, the Xenon BrightStar (D.O.R.C., Zuidland, The Netherlands) has adjustable filters that filter light below 420 nm, 435 nm, 475 nm, and 515 nm. With this, a surgeon can decrease the hazard to the retina and change the CCT inside the eye. In mercury vapor light sources, an integrated 435 nm filter is included, e.g., Photon II (Synergetics Inc., O’Fallon, MO, USA). The xenon light source Photon (Synergetics Inc., O’Fallon, MO, USA) provides a 485 nm filter, and the xenon light source Stellaris PC (Bausch & Lomb, Rochester, NY, USA) also features three types of filters (green-tin, yellow-tin, and amber filters) to reduce retinal hazard. However, when applying these filters, the CCT would be lower, and the intraocular light would be red shifted. In diaphanoscopic illumination, this would lead to a much stronger red shift. To compensate for this red shift, the blue wavelength component of the emission spectrum of the light source would have to be increased again. However, caution must be taken to ensure that it does not drastically increase photochemical hazard to the retina, as blue light can contribute to retinal damage [50,51,52,53,54]. Increasing the blue light content in compliance with the limits for photochemical hazard to the retina must be ensured according to the DIN EN ISO 15004-2 [54]. This increase in blue light content, the resulting CCT increase, and the corresponding photochemical hazard will be investigated in the future.

## 5. Conclusions

In this study, correlated color temperature in ex vivo porcine eyes is determined during diaphanoscopic illumination and endoillumination. A strong red shift of the illumination light during diaphanoscopic illumination is demonstrated. As surgeons are used to white light in endoillumination application, the correlated color temperature should be high and, therefore, the red shift should be compensated, e.g., by increasing the blue component of the emission spectrum of the light source. This should be considered in the development of new diaphanoscopic illumination systems. However, the retinal risk should also be taken into account.

## Figures and Tables

**Table 1:** Direct comparison of correlated color temperature (CCT) of the light emission in front of an ophthalmological fiber in combination with a halogen light source and a xenon light source to the CCT inside the eye during endoillumination and with diaphanoscopic illumination.

CCT (K)	Fiber Emission	Endoillumination	Diaphanoscopy
Halogen lamp	3885 ± 62	3791 ± 213	2129 ± 134
Xenon lamp	5281 ± 100	5182 ± 406	2567 ± 197

## Data Availability

The data presented in this study are available from the corresponding author upon request.
